# Research fatigue among injecting drug users in Karachi, Pakistan

**DOI:** 10.1186/1477-7517-10-9

**Published:** 2013-06-11

**Authors:** Aysha Zahidie, Arshad Altaf, Adeel Ahsan, Tanzil Jamali

**Affiliations:** 1 Department of Community Health Sciences, The Aga Khan University, Karachi, Pakistan; 2 Sind AIDS Control Program, Canada-Pakistan HIV/AIDS Surveillance Project, Karachi, Pakistan

**Keywords:** HIV, IDUs, Research fatigue

## Abstract

**Background:**

Karachi is the largest metropolis of Pakistan and its economic hub attracting domestic migrants for economic opportunities. It is also the epicenter of HIV epidemic in the country. Since 2004, one pilot study and four behavioral and biological surveillance rounds have been conducted in Karachi. In addition many student research projects have also focused on key risk groups including injection drug users (IDUs). As a result of this extra ordinary exposure of same kind of questions, IDUs know how to respond to high value questions related to sharing of needles or unsafe sexual practices. The purpose of the study was to explore the element of research fatigue among IDUs in Karachi, Pakistan.

**Methods:**

The study was conducted on 32 spots in Karachi, selected on the basis of estimate of IDUs at each spot. A trained field worker (recovered IDU) visited each spot; observed sharing behavior of IDUs and asked questions related to practices in January 2009. Verbal consent was obtained from each respondent before asking questions.

**Results:**

On average 14 IDUs were present at each spot and out of 32 selected spots, 81% were active while more than two groups were present at 69% spots. In each group three to four IDUs were present and everyone in the group was sharing. One dose of injecting narcotics was observed. Sharing of syringes, needles and distilled water was observed at 63% spots while professional injector/street doctor was present at 60% spots.

**Conclusion:**

There is a need to check internal consistency in surveillance research. It is highly likely that IDUs and other risk groups know how to respond to key questions but their responses do not match with the practices.

## Background

Among low and middle income countries, HIV epidemic is presenting itself with a specific geographical drift, as it is spreading from major urban cities and provincial capitals to smaller cities and towns [1]. Among the many factors, the most important one contributing to this trend is unsafe drug injecting practices. Injection drug user (IDU) share contaminated syringes to quantify or mix drug preparations and accessories (e.g. cotton, distilled water and/or ampoules) which are common risk factors of HIV AIDS transmission in low and middle income countries [2].

Karachi is the largest city of Pakistan with an estimated population of around 13 million persons. As the financial hub of the country it has been facing a burden of in country migration from all over Pakistan for economic opportunities. It is a sprawling metropolis with multiple slum areas (*kachiabadis*) and peri-urban localities. As a mega city, it has a huge challenge to provide health and other services to the population influx. The National AIDS Control Program reports that there are more than 16,000 IDUs in 18 towns of Karachi. In 2011, HIV prevalence among IDUs in Karachi was found to be 42%. While studies conducted in other major cities of Pakistan reported variable prevalence of HIV among IDUs as 25.4% in Hyderabad, 19.2% in Sukkur and 52% in Sargodha [3-5].

IDUs have been part of multiple rounds of surveillance in Karachi. Since 2004 one pilot study and four surveillance rounds have been conducted. Moreover, many student research projects have also focused on IDUs. The focus of these projects have been on issues like sharing of needles, unsafe sex etc [6-8].

In 2004 the first pilot study was conducted in Karachi and Rawalpindi which showed 23% (94/402) of IDUs in Karachi were HIV infected. Forty eight percent IDUs reported sharing a used needle in the previous week and 6% did so for all injections [6]. In subsequent surveillance rounds while the prevalence of HIV increased, the reported improvement in practices was also noticed. For example HIV surveillance round in 2006–7 indicated that sharing of last injection was 9% while practice of always using a new syringe was 82%. However prevalence of HIV among this group rose to 30% [7]. In 2008, round III of surveillance reported that sharing of last injection was 23% while practice of always using a new syringe was 45% and HIV prevalence came down to 23% [8].

This mismatch in self-reported practices and actual prevalence of HIV among IDUs was intriguing and of concern. It is believed that as a result of being part of multiple research activities IDUs now know how to answer questions related to their risky behaviors for example questions about sharing of syringes and needles or unsafe sexual practices. The responses of IDUs on high value questions are erratic at best indicating the need to address this important issue. We conducted this study in order to determine whether responses of IDUs match with their practices.

## Material and methods

A cross sectional study was conducted in Karachi, Pakistan in January 2009. Data was collected by a trained outreach worker previously engaged in surveillance research. A total of 36 hot spots of IDU in Karachi were selected based on the number of estimates.

**Figure 1:**
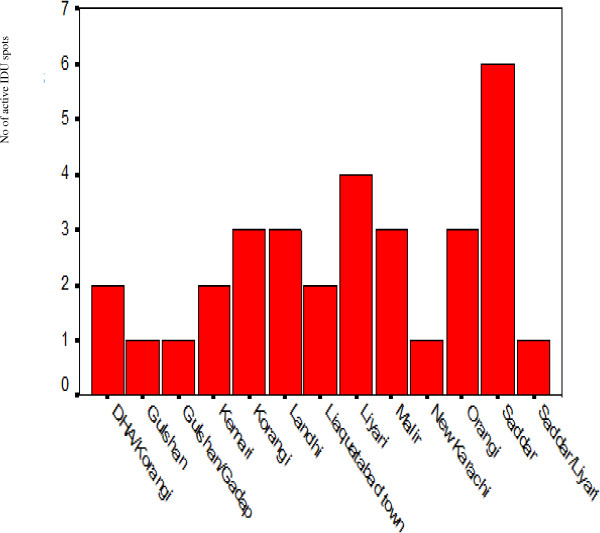
Active IDU sites in various towns of Karachi.

A hot spot is defined as a place/location where risk group for HIV are present and indulge in unsafe practice or behavior that can expose them towards HIV infection. The selected spots had the highest number of estimates among the 808 spots mapped prior to the study. The field worker who was familiar with the city and surroundings was provided the list of hot spots and transportation (Figure 1). The selected hot spots had more than 15 IDUs present. The field worker introduced himself and explained to the IDUs that he is conducting a study to determine behaviors and if they are willing to participate he will ask a few questions. He asked them their age, educational status and where they were living presently. The other questions were related to their drug injecting behaviors including the number of narcotic injections taken per day. The other questions were observations which included:

● Number of IDUs present at the spot

● If in group how many groups were at the hot spot

● If more than one group how many IDUs were there in each group

● Drugs mainly injected

● Injecting paraphernalia used and whether it was shared e.g. cotton, cloth, cookers, spoons, bottle caps

● Preparation of drug

● Number of IDUs with their own syringe

● Presence of “street doctor” at the hot spot and whether he was using new or used syringes while injection the drug users

For the purpose of this study we used following case definitions:

IDU: Any male injecting drugs for non-therapeutic reasons for six months.

Street doctor: A drug peddler who provides drugs to addicts and is an expert in helping IDUs in finding veins in order to inject drugs.

IDU groups: IDUs gathered at any spot in more than two in numbers.

Inclusion criteria: All spots where more than two IDUs meeting our case definition were present and who consented for this study were included.

Exclusion criteria: IDUs who were intoxicated or those who refused to consent were excluded.

Data collection tools: Observation + brief interview questionnaire.

### Statistical analysis

Data were analyzed using Statistical Package for Social Sciences (SPSS) version 19.

Descriptive analysis was done by calculating mean (+standard deviation) for continuous and percentages for categorical variables on SPSS vs. 19.

### Ethical review

The study was ethically reviewed and approved by ethical review committees of Health Canada in Canada and HOPE (NGO) in Pakistan.

## Result

The results showed that out of 32 selected spots, 81% were active while more than two groups were present at 69% spots. Highest number of hot spots was in Sadder Town followed by Lyari and Orangi towns (Table 1). On average 14 IDUs were present at each spot. 98% IDUs were males and average age was 30.4 years ± 8.0. Most of the IDUs were illiterate (57%) and 48% were living on the street. Largest number of IDUs was found in Liyari town followed by Landhi (Table 1). Sharing was observed at 62.5% spots whereas sharing of syringes, needles and distilled water was observed at 53.1% spots (Table 2). One dose of taking the narcotic injection was observed in this study.

**Table 1:** No of IDUs present at various spots of different towns visited in Karachi

**Town**	**Spot**	**No.of IDUs**	**Total IDUs per town**
DHA/Korangi	QayyumabadGali no. 18	8	28
	*Kala pul*	20	
Gulshan	Civic Center under fly over	11	11
Gulshan/Gadap	Sohrab Goth bridge	7	7
Kemari	Masan Rd. near railway *phatak*	24	28
	KPT Ground	4	
Korangi	Bismillah *2*.*5 no*. *stop *Korangi	4	4
	Ibrahim Hyderi	0	
	100 quarter Korangi	0	
Landhi	Qaidabad Flyover (under it)	18	60
	Railway lines near Qaidabad stations	17	
	Rehri Goth	25	
Liaquatabad town	Moosa Colony near Ziauddin Hospital	6	26
	Petrol Pump chowrangi under the flyover	20	
Liyari	Liyari Football stadium opposite Liyari General Hospital	100	162
	Meera Naka near Water Board Pumping station	38	
	Ali Bagh graveyard Nayabad	16	
	Kingri ground Liyari	8	
Malir	Kalaboard*Bhangi Para* Sahib dad Goth	0	30
	Saudabad graveyard Sabir Colony	17	
	Gharibabad D6 Stop	13	
New Karachi	60 no Bus Stop	35	35
Orangi Town	Marhaba Bakery	2	2
	*KachiAbadi*	0	
	*Kati Pahari*	0	
Saddar	Ramswami tea shop on main road	8	21
	Civil Hospital	7	
	Burns Rd. behind Sindh Secretariat	0	
	Radio Pakistan	0	
	Saddar hotel near United Bakery	6	
	Preddy police station	0	
Saddar/Liyari	Kharadar Hospital Opposite MoosaKabari	22	22

**Table 2:** Observation of IDU characteristics at 32 spots

**Characteristics**	**Yes n (%)**	**No n (%)**
Spot active	26 (81.3)	6 (18.8)
Sharing observed or not	20 (62.5)	12 (37)
Sharing syringes only	3 (9.4)	29 (90)
Sharing syringes, needles and distilled water	17 (53.1)	15 (46.9)
Two or more groups present	22 (68.8)	109 (31.3)
More than two IDUS per group	23 (71.9 )	9 (28.1)
All groups sharing	20 (62.5)	12 (37.5)
Street doctor present or not	19 (59.4)	13 (40.6)

On verbal inquiry it was told that on average each IDU injected 2.9 ± 1.4 injections per day and 91% injected in parks/streets while 81% injected in groups as well. 70.3% were injected by “professional injectors” during past month. Out of these, 12.2% reported always getting their injections from these professional injectors. 38.6% IDUs told that they always injected with a new needle while 31.2% used someone else’s needle/syringe at last injection and 23% of IDUs passed on needle/syringe to another IDU as well. Condom used at last sexual encounter was reported by 25.8% and 56% told that they had never heard of any preventive programs. Presence of professional injector/street doctor was also observed at 60% spots.

## Discussion

IDUs have been the target of public health interventions and also the focus of epidemiological research because of the imminent threat of the spread of HIV. A high proportion of IDUs (70%) in our study utilized services of “street doctor” and extremely high number (81%) inject in groups. These practices expose them to risk of acquiring HIV and other infections. This issue should be kept in mind when conducting future research in order to get correct and reliable response which could reflect on the quality of services provided to them and also the quality of outreach workers who are assigned to improve their risky behaviors.

Socio demographic characteristics of IDUs in seven cities of Pakistan were explored in National HIV Surveillance Rounds Reports showing that only 16% were living on streets. In contrast to it, our results showed that about 48% IDUs were living on streets. It could be due to the different demographics of other cities of Pakistan [7,8].

IDUs living on street are at greater risk for infections related to injection drug use, including abscesses, septicemia, endocarditis and tuberculosis. Co-infection with hepatitis C and/or B is extremely common. The reason of this higher risk is because of limited access to existing health services due to low literacy levels or fear of discrimination by health care providers [9]. While our result indicate that 70.3% were injected by “professional injectors” during past month another study has reported that 7.7% IDUs in Karachi and 22.8% in Sargodha used help of professional injectors [5] thus confirming the pattern of this practice. Fairbairn et al. have already described higher HIV risk associated with street injectors as providing help in injecting was associated with various high-risk behaviors, including elevated levels of syringe lending [10]. In round III most IDUs reported that they injected most recently in open spaces/streets/parks and were accompanied by friends and acquaintances (64.1%). 32% of IDUs injected alone, 16.2% injected in Shrines and *darbars*, while 2.3% injected in company with other family members [7,8]. Literature has already shown that size and density of sharing networks are key determinants of transmission of HIV and STIs. A similar dynamic may operate for syringe sharing networks, particular when large groups of IDUs sit together and inject [11-14]. Our observations warrant more detailed study of how these networks form and operate.

There are several limitations of this assessment. This is a brief assessment of the situation and did not use a scientifically rigorous sampling frame. However, our study sampled a large proportion of the IDUs in the city. Secondly, we did not study the network structure of the injecting community. Our recruitment process may have selected for IDUs that were staying at the injecting sites and we were able to observe one dose of injecting narcotics. However with all these limitations this research highlights the importance of ongoing research to inform program implementer as different drug-using behaviors pose different risks for HIV transmission.

At an individual level, interventions aim to change behavior to reduce HIV risks, with the ultimate goal of risk elimination. Specific interventions as mentioned in literature could be:

● HIV information, education and communication (IEC) programs

● Risk-reduction counseling

● Voluntary counseling and HIV testing (VCT)

● Disinfection programs

● Needle-syringe programs

● Agonist pharmacotherapy programs

● HIV treatment and care [15,16]

## Conclusion

It is important to consider and address the specific risks and vulnerabilities of IDUs due to high risk behaviors improving their access and utilization of appropriate services. Moreover it is of utmost importance to get correct and reliable response which could reflect on quality of services provided to IDUs and also the quality of outreach workers who are assigned to improve their risky behaviors.

## Competing interests

The authors declare that they have no competing interests.

## Authors’ contributions

AA conceived the study and supervised the data collection. AZ, AdA and TJ, performed the statistical analysis. AA, AZ, AdA and TJ drafted and revised the manuscript. All authors read and approved the final draft.
